# Bone marrow mesenchymal stem cells inhibit hepatic fibrosis via the AABR07028795.2/rno-miR-667-5p axis

**DOI:** 10.1186/s13287-022-03069-7

**Published:** 2022-07-28

**Authors:** Yuan Feng, Yanjie Li, Mingxing Xu, Hongyu Meng, Cao Dai, Zhicheng Yao, Nan Lin

**Affiliations:** 1 Department of Hepatobiliary Surgery, The Third Affiliated Hospital of Sun Yat-Sen University, No. 600, Tianhe Road, Tianhe District, Guangzhou, 510630 Guangdong China; 2 Department of General Surgery, The Third Affiliated Hospital of Sun Yat-Sen University, Guangzhou, 510630 Guangdong China

**Keywords:** Hepatic fibrosis, BMSCs, Hepatic stellate cells, lncRNA, miR-667-5p

## Abstract

**Background:**

The mechanism of bone marrow mesenchymal stem cells (BMSCs) in treating hepatic fibrosis remains unclear.

**Methods:**

TGF-β1-induced hepatic stellate cell (HSC)-T6 and CCl4-induced hepatic fibrosis rats were treated with BMSCs. HSC-T6 cell activity was determined using the cell counting kit-8 assay, and the histology change was evaluated using hematoxylin and eosin and Masson staining. The expression of fibrosis markers was determined using real-time quantitative PCR, Western blotting, and immunocytochemistry. RNA sequencing (RNA-seq) was used to screen the lncRNAs involved in the effect of BMSCs in fibrosis, and the function of fibrosis-associated lncRNA in fibrosis histology change and fibrosis marker expression was investigated. The potential miRNA target of lncRNA was predicted using R software. The interaction between lncRNA and miRNA was verified using luciferase report system and RNA immunoprecipitation (RIP) in 293T and HSC-T6 cells.

**Results:**

BMSC attenuated TGF-β1-induced HSC-T6 activation and suppressed the expression of fibrosis-associated gene (MMP2, Collagen I, and αSMA) expression at the transcription and translation levels. BMSC treatment also improves hepatic fibrosis in rats with CCl4-induced fibrosis by decreasing the expression of fibrosis-associated genes and suppressing collagen deposition in the liver. RNA-seq revealed that AABR07028795.2 (lnc-BIHAA1) was downregulated in the TGF-β1-induced HSC-T6 after treatment with BMSCs as compared with those in TGF-β1-induced HSC-T6, and subsequently, functional analysis showed that lnc-BIHAA1 plays a beneficial role in suppressing hepatic fibrosis. Luciferase activity assay and RIP revealed that lnc-BIHAA1 interacted with the miRNA, rno-miR-667-5p, functioning as a fibrosis phenotype suppressor in TGF-β1-induced HSC-T6. Moreover, overexpression of rno-miR-667-5p significantly reverses the effect of lnc-BIHAA1 on HSC-T6.

**Conclusions:**

BMSC treatment suppresses hepatic fibrosis by downregulating the lnc-BIHAA1/rno-miR-667-5p signaling pathway in HSCs. Our results provide a scientific basis for establishing BMSCs as a biological treatment method for liver fibrosis.

## Introduction

Hepatic fibrosis is a typical response to various chronic liver injuries causing viral hepatitis and alcohol toxicity, which results in the overproduction of extracellular matrix (ECM), ultimately leading to an imbalance between the production and dissolution of ECM [1]. Regarding cells in the liver, hepatic stellate cells (HSCs) are the dominant collagen-producing cells and play a pivotal role in the occurrence and progression of hepatic fibrosis [2]. Therefore, the activation and proliferation of HSCs are considered key events in the process of hepatic fibrosis. A therapeutic strategy based on suppressing HSC activation and proliferation theoretically reduces the development and progression of hepatic fibrosis [3].

Over the past decades, numerous studies have contributed their efforts to search for therapy for hepatic fibrosis. Cell therapy, especially mesenchymal stem cell therapy, is one of the promising therapies. Since stem cells are easily isolated from the bone marrow, they have low immunogenicity and have high self-renewal and pluripotent differentiation potential, which has practical advantages in tissue regeneration [4–7]. Several animal experiments have shown that bone marrow mesenchymal stem cell (BMSC) treatment has the effects of anti-liver fibrosis, improving symptoms and liver function, suggesting the effectiveness and safety of BMSCs in clinical applications. However, its internal regulation mechanism is still unclear.

lncRNA is a class of noncoding RNA with a size of more than 200 nucleotides [8]. Increasing studies have suggested that lncRNAs are frequently dysregulated in various physiological and pathological processes and involved in cancer initiation and progression [9], metabolic diseases [10], and inflammation disease [11, 12]. Emerging evidence suggests that lncRNAs are involved in cellular mechanisms and therapeutic targets to treat hepatic fibrosis. By regulating the pyroptosis of macrophages [13], activating the classic pro-fibrosis pathway [14], or acting as a miRNA sponge, it exerts an anti-fibrosis function by inhibiting the miRNA-mediated fibrosis pathway [15–18]. However, the role and biological mechanisms of the vast majority of lncRNAs in the development and progression of hepatic fibrosis are still largely unknown. In addition, the involvement of lncRNA in treating BMSCs also needs to be studied further.

In this study, we analyzed the anti-fibrosis effect of BMSCs in both TGF-β1-induced liver stellate cell lines and rats with hepatic fibrosis phenotype. Further, we analyzed the biological mechanism underlying the BMSC treatment from the perspective of the lncRNA–miRNA interaction. Our findings might provide evidence for BMSCs in treating hepatic fibrosis and add new knowledge of the role of the lncRNA/miRNA axis in the process of hepatic fibrosis.

## Materials and methods

### Cell culture and BMSC treatment

A rat liver stellate cell line, HSC-T6, a human endothelial kidney cell line, HEK293T, and BMSCs were used. HSC-T6 cell lines were purchased from Procell Life Science and Technology Co., Ltd. (Wuhan, China). BMSCs were purchased from Guangzhou SALIAI Stem cell Science and Technology Co., Ltd. HEK293T cell lines were purchased from the National Collection of Authenticated Cell Cultures. HSC-T6 and HEK293T cells were maintained in Dulbecco’s modified Eagle’s medium (DMEM; Gibco, USA) with high glucose (4500 mg/L), 10% fetal bovine serum (Gibco), 100 U/mL penicillin, and 100 g/mL streptomycin (Gibco) and cultured in an incubator containing 5% CO_2_ at 37 °C. BMSCs were maintained in a stem cell-specific medium purchased from Guangzhou SALIAI Stem cell Science and Technology Co., Ltd. (SALIAI, G03010). For TGF-β1 induction, HSC-T6 cells were induced with TGF-β1 (2 or 10 ng/ml) for 24 h. For evaluating the therapeutic effects of BMSC treatment, HSC-T6 cells were grown in the lower chamber of a transwell system and induced with TGF-β1 (10 ng/ml) before co-culture. After 24 h of induction, BMSCs were seeded on the upper chamber and co-cultured with HSC-T6 for 72 h.

### Hepatic fibrosis rat model construction and BMSC treatment

Rats (male SD rats, weighing approximately 130 ± 10 g) were purchased from the Guangdong Medical Experimental Animal Center (Guangzhou, China). Hepatic fibrosis rats were induced with CCl4 (1 mL/kg, CCl4: olive oil, v/v = 1:1) using intraperitoneal injections twice each week over 8 weeks. Referring to the previous studies [19, 20], the effect of BMSC treatment on hepatic fibrosis rats was evaluated by injecting the BMSCs (1 × 106 cells per rat) into the right lobe of the liver. After BMSC treatment, rats continue to feed for 3 weeks. Hepatic fibrosis rats without BMSC treatment/injected with PBS control were used as a sham and negative control. To investigate the effect of a knockdown on the lnc-BIHAA1 on hepatic fibrosis, lentivirus-based sh-lnc-BIHAA1 and control vectors were constructed and packaged (GenePharma, Shanghai, China) according to standard protocols. Two weeks after the CCl4 injection mentioned above, the resultant sh-lnc-BIHAA1 and control virus (6 × 107 TU/rat/200 μl) was injected into rats through the tail vein. At week 11, the liver tissue was collected for subsequent experiments. This experiment was approved by the ethical committee of Guangzhou Forevergen Biosciences (Guangzhou, China).

### Cell counting kit-8 (CCK-8) assay

Approximately 4 × 10^3^ cells were cultured in a 96-well plate before the CCK-8 assay. Cells that received corresponding treatment continued to culture for 24, 48, and 72 h. CCK-8 reagent (Beyotime) was added to cells according to the manufacturer’s instructions for cell activity measurement. The absorbance value at 450 nm on a microplate reader was recorded (BioTek, Vermont, USA).

### Western blotting (WB)

Total proteins were extracted from HSC-T6 cells by RIPA (Beyotime, Shanghai, China) supplemented with phenylmethylsulfonyl fluoride. The proteins were separated with sodium dodecyl sulfate-polyacrylamide gel electrophoresis and then transferred onto the PVDF membranes. After blocking with 5% defatted milk powder, the membranes were incubated with primary antibodies of MMP2 (1:1000, Abcam, ab97779), anti-alpha smooth muscle (α-SMA, 1:1000, Boster, bm0002), collagen I (1:1000, Proteintech, 14695-1-AP), and GAPDH (1:2000, Proteintech, 60004-1-lg) at 4 °C overnight. The next day, membranes were incubated with secondary horseradish peroxidase-labeled secondary antibody (1:5000, Zhongshan Biotechnology, Beijing, China) at room temperature for 1 h. An enhanced chemiluminescence detection system (GE Health Care, Waukesha, USA) was used to visualize the proteins. The relative expression of proteins was normalized to that of the internal control GAPDH.

### Real-time quantitative PCR (qRT-PCR)

Total RNA was extracted from HSC-T6 cells or liver tissue using TRIzol reagent (Invitrogen, USA). cDNA was synthesized using Prime Script RT reagent kit (Takara, Tokyo, Japan). Power SYBR Green PCR Master Mix (Bio-Rad) was used for gene amplification according to the manufacturer’s instructions. qRT-PCR was performed on an ABI7500 Real-Time PCR System, and the relative gene expression was calculated using the 2^−ΔΔCT^ method. GADPH was used as an internal control. The primers used in this study are shown in Table 1.

**Table 1:** Primers and oligonucleotides used in this study

Primer name	Sequence (5’-3’)
AABR07069008.3-F	CTGCCAGCAACCATCAGTTA
AABR07069008.3-R	AGGTTAGGGAGGAGCACCAT
LOC103692471-F	AGGTGACTCAGCCTGCTTGT
LOC103692471-R	GGGACTTTCCCTACGATTCC
AC095078.2-F	GAGAGGTCCGGTCTGACTTG
AC095078.2-R	TGTCAACTTGCACACCCATT
AC139608.2-F	ATGACTGACGTCCCTGGTTC
AC139608.2-R	CCAAGTGCTGGGGTTACTGT
AABR07028795.2-F	CACTTTCTGCCCCAGTTCAT
AABR07028795.2-R	TCTTGCTTCTGCATTTGGTG
Rat-α-SMA-F	ACCATCGGGAATGAACGCTT
Rat-α-SMA-R	CTGTCAGCAATGCCTGGGTA
Rat-COL1(Collagen I)-F	GGAGAGAGCATGACCGATGG
Rat-COL1(Collagen I)-R	GGGACTTCTTGAGGTTGCCA
Rat-Mmp2-F	AGCTTTGATGGCCCCTATCT
Rat-Mmp2-R	GGAGTGACAGGTCCCAGTGT
Rat-GAPDH-F	GCAAGAGAGAGGCCCTCAG
Rat-GAPDH-R	TGTGAGGGAGATGCTCAGTG
Si-Rat-AABR07028795.2-1	GCTTGAGCAGCACTGACTA
Si-Rat-AABR07028795.2-2	GCACAGATCCACAGGTCTA
Si-Rat-AABR07028795.2-3	CCTGAGAAATCGTAGCCTT
siRNA-NC	UUCUCCGAACGUGUCACGUTT
Rno-miR-667-5p mimics (5’-3’)	CGGUGCUGGUGGAGCAGUGAGCAC
	GCUCACUG CUCCACCAGCACCGUU
Rno-miR-667-5p inhibitor (5’-3’)	GUGCUCACUGCUCCACCAGCACCG
miRNA mimics NC (5’-3’)	UUCUCCGAACGUGUCACGUTT
	ACGUGACACGUUCGGAGAATT
miRNA inhibitor NC (5’-3’)	CAGUACUUUUGUGUAGUACAA

### Immunohistology assay

Paraffin-embedded sections of 5 µm thickness were dehydrated and stained with hematoxylin and eosin (HE) and Alcian blue. To evaluate the expression of MMP2, collagen I, and α-SMA, the sections were placed in an antigen repair solution buffer (pH 9.0) and then blocked in 2% normal goat serum. Afterward, sections were incubated with primary antibodies, including anti-MMP2 antibody (Abcam), α-SMA (Boster), and collagen I (Proteintech) antibody. Signals were detected using the VECTASTAIN ABC Elite Kit (Vector Laboratories, CA, USA), followed by DAB Substrate Kit (Vector Laboratories) according to the manufacturer’s instructions.

### Hematoxylin and eosin (HE) and Masson’s trichrome staining

Liver tissue sections of 5 μm thickness were separately stained with hematoxylin and eosin (HE) and Masson’s trichrome. The morphological changes and collagen content were observed using microscopy (Nikon 80i, Tochigi, Japan).

### RNA sequencing (RNA-seq) and lncRNA expression analysis

To draw lncRNA expression profiles, total RNA was qualified and fragmented. A cDNA library was prepared using the SmartPCR cDNA kit (CLONTECH Laboratories, Japan) and subjected to RNA-seq for lncRNA using an Illumina HiSeq2000 perform (BGI-Shenzhen, Shenzhen, China). To analyze the differential expression of lncRNA, raw data were mapped to a rat reference genome, and the differentially expressed lncRNAs were identified using Cufflinks v.2.0.2 with default parameters. The significantly different expressed lncRNAs were defined by the following criterion: |log2Ratio|≥ 0.67, *P* value < 0.05. The potential function of differentially expressed lncRNAs and possible pathways that might be involved was predicted using the Gene Ontology (GO) and Kyoto Encyclopedia of Genes and Genomes (KEGG) platform (http://www.genome.jp/kegg/) analyses using R package. The lncRNA–miRNA interaction was predicted by the R package and visualized by Cytoscape software.

#### *Terminal deoxynucleotidyl transferase dUTP nick end labeling* (*TUNEL assay) and immunofluorescent staining*

TUNEL assay was conducted similar to the immunohistology assay. Sections were stained according to the protocol of A Click-iT TUNEL Alexa Fluor 488 Imaging Assay (Invitrogen) and incubated with the primary anti-desmin (1:100, Proteintech, #16520-1-AP) with a subsequent incubating with corresponding secondary antibody (1:1000, Invitrogen, #A11012). Cell nuclei were stained with DAPI. Fluorescent images were observed under a fluorescence microscope (Olympus, Tokyo, Japan).

### *Fluorescent *in situ* Hybridization (FISH)*

FISH was performed using the Fluorescent in situ Hybridization Kit according to the manufacturer’s instructions (Thermo Fisher, Shanghai, China). Cells cultured on coverslips (18-mm round) were fixed with 4% formaldehyde and treated with PBS containing 0.5% Triton X-100. After blocking, cells were incubated in hybridization solutions (2 × SSC, 100 mg/ml dextran sulfate, and 10% formamide) containing AABR07028795.2-specific 6-FAM-labeled probe (BersinBio, Guangdong Province, China) at 37 °C overnight in a humidified chamber. The slides were washed with 4 × SSC, 2 × SSC, and 1 × SSC for 5 min each. Finally, the coverslips were counterstained with DAPI. The signal was observed under fluorescence microscopy.

### Hyaluronic acid (HA), hydroxyproline (HYP), and liver function indicators measurement

For liver tissues HYP and HA measurement, the kits for HYP (#A030-2-1; Jiancheng Bioengineering Institute, Nanjing, China) and HA (#H141-1-2; Jiancheng Bioengineering Institute) were used. The serum levels of liver function indicators including alanine aminotransferase (ALT), aspartate aminotransferase (AST), total bilirubin (TBIL), and albumin (ALB) were determined by kit from Jiancheng bioengineering Institute (corresponding product number: #C009-2-1, #C010-2-1, #C019-1-1, and #A028-2-1) following the manufacturer’s instructions.

### Cell transfection

HSC-T6 cells (5 × 10^5^ cells) were seeded on 6-well plates before transfection. The cell medium was replaced with serum and antibiotic-free DMEM. siRNAs targeting AABR07028795.2, rno-miR-667-5p mimic/inhibitor (GenePharma, Shanghai, China), and AABR07028795.2 overexpressing vector (GENERAL BIOL, Chuzhou, China) were transfected into cells using Lipofectamine 2000 transfection reagent (Invitrogen, MA, USA). Scramble siRNA/miRNA mimic/inhibitor and an empty vector were used as a negative control. The sequence of siRNA, miRNA mimics, and miRNA inhibitors is listed in Table 1.

### Luciferase activity assay

Wild-type (WT) or mutated fragments of AABR07028795.2 were subcloned into the luciferase reporter plasmid pmirGLO luciferase vector (Promega, USA), resulting in the WT (AABR07028795.2 WT) and mutant (MUT) plasmids (AABR07028795.2 MUT1and AABR07028795.2 MUT2). The WT and MUT plasmids were co-transfected with rno-miR-667-5p mimics or miRNA mimic NC. After 24 h, luciferase activity was determined using the dual-luciferase reporter assay system (Promega, USA).

### RNA immunoprecipitation (RIP) assay

The relationship between AABR07028795.2 and rno-miR-667-5p was analyzed using the Magna RIP RNA-Binding Protein Immunoprecipitation Kit (Millipore, USA). The co-precipitated RNAs were purified and transcribed to cDNA for qRT-PCR analysis. Primers specific for AABR07028795.2 and rno-miR-667-5p were used for qPCR. Antibodies used in RIP assay included anti-AGO2 and control IgG (Millipore, USA).

### Statistics

Data were presented as mean ± standard deviation (SD). The data were analyzed using GraphPad Prism software v.7.0 (GraphPad Software, San Diego, CA, USA). Differences between groups were analyzed using Student’s *t* test. A one-way or two-way analysis of variance was used for the comparison of more than three groups. *P* < 0.05 was considered statistically significant.

## Results

### BMSC treatment attenuates fibrotic phenotype of TGF-β1-induced HSC-T6 cells and hepatic fibrosis rats

**Fig. 1:**
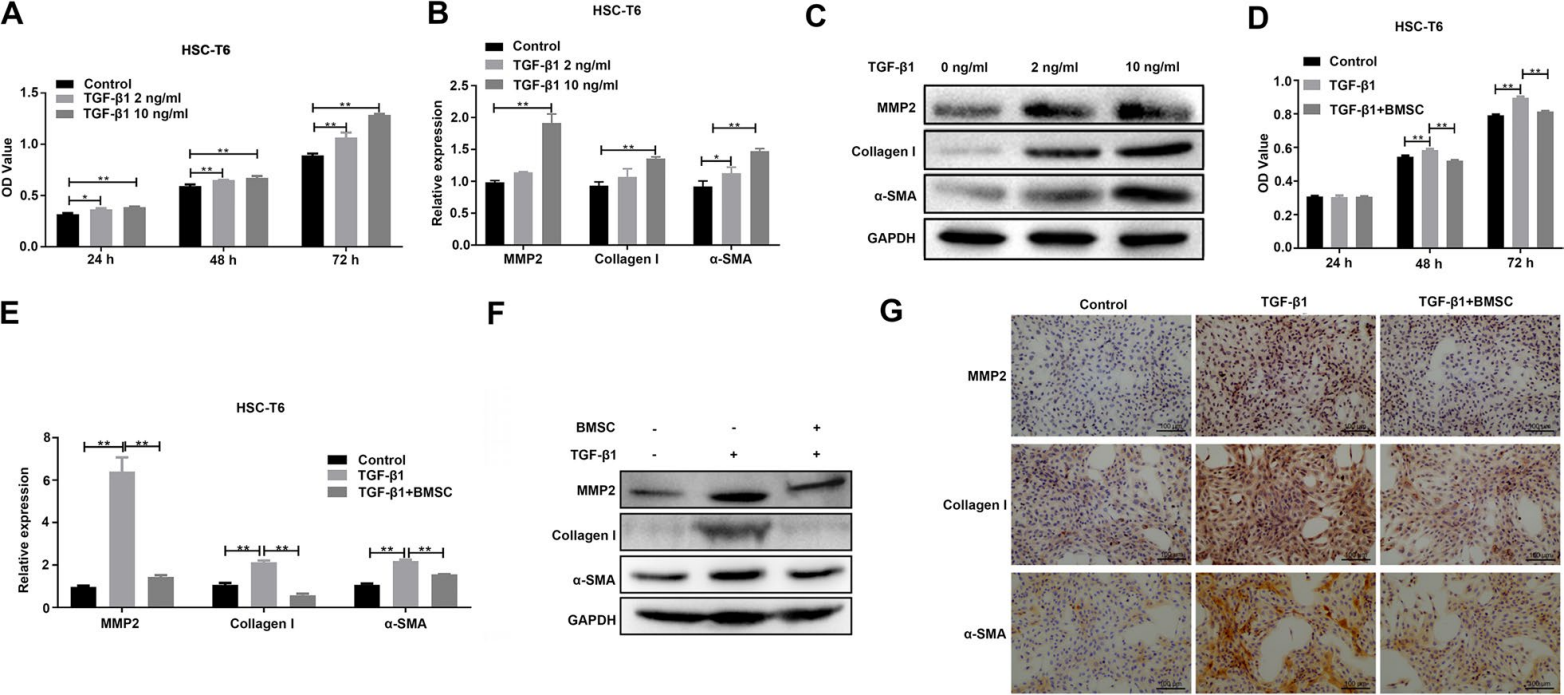
Effects of BMSC treatment on the activation of HSC-T6 induced by TGF-β1. **A** The effect of TGF-β1 on HSCT activation detected by CCK-8 assay at 24, 48, and 72 h after treatment. qRT-PCR (**B**) and WB (**C**) results showing the effects of induction on the expression of fibrotic phenotype-associated genes, MMP2, collagen I, and α-SMA. **D** CCK-8 assay showing the effects of BMSC treatment on HSC-T6 cell activation induced by TGF-β1. qRT-PCR (**E**) and WB (**F**) assays show BMSC treatment’s effect on the expression of fibrotic phenotype-associated gene induced by TGF-β1. **G** IHC assay showing the reverse effect of BMSC treatment on the fibrotic phenotype-associated gene induced by TGF-β1

In order to study the effect of TGF-β1 on the proliferation and fibrotic phenotype of HSC-T6 cells, cells were treated with different concentrations of TGFβ1 (2 ng/mL and 10 ng/mL). CCK-8 assay was performed to detect cell viability at 24, 48, and 72 h after TGF-β1 treatment. It was found that TGF-β1 stimulation significantly increased the cell viability of HSC-T6 cells in a concentration-dependent manner, and as time increased, cell viability increased (Fig. 1A). qRT-PCR and WB showed that the fibrosis marker, MMP2, a-SMA, and collagen I mRNA and protein expression increased HSC-T6 cells treated with TGF-β1 for 24 h in a concentration-dependent manner (Fig. 1B and C). However, when HSC-T6 cells were co-cultured with BMSCs, BMSC treatment significantly inhibited the proliferation of HSC-T6 cells (Fig. 1D). Simultaneously, BMSC treatment for 72 h significantly inhibited the upregulation of mRNA and protein expression of MMP2, a-SMA, and collagen I induced by TGF-β1, as evidenced by qRT-PCR, WB, and IHC assays (Fig. 1E–G).

**Fig. 2:**
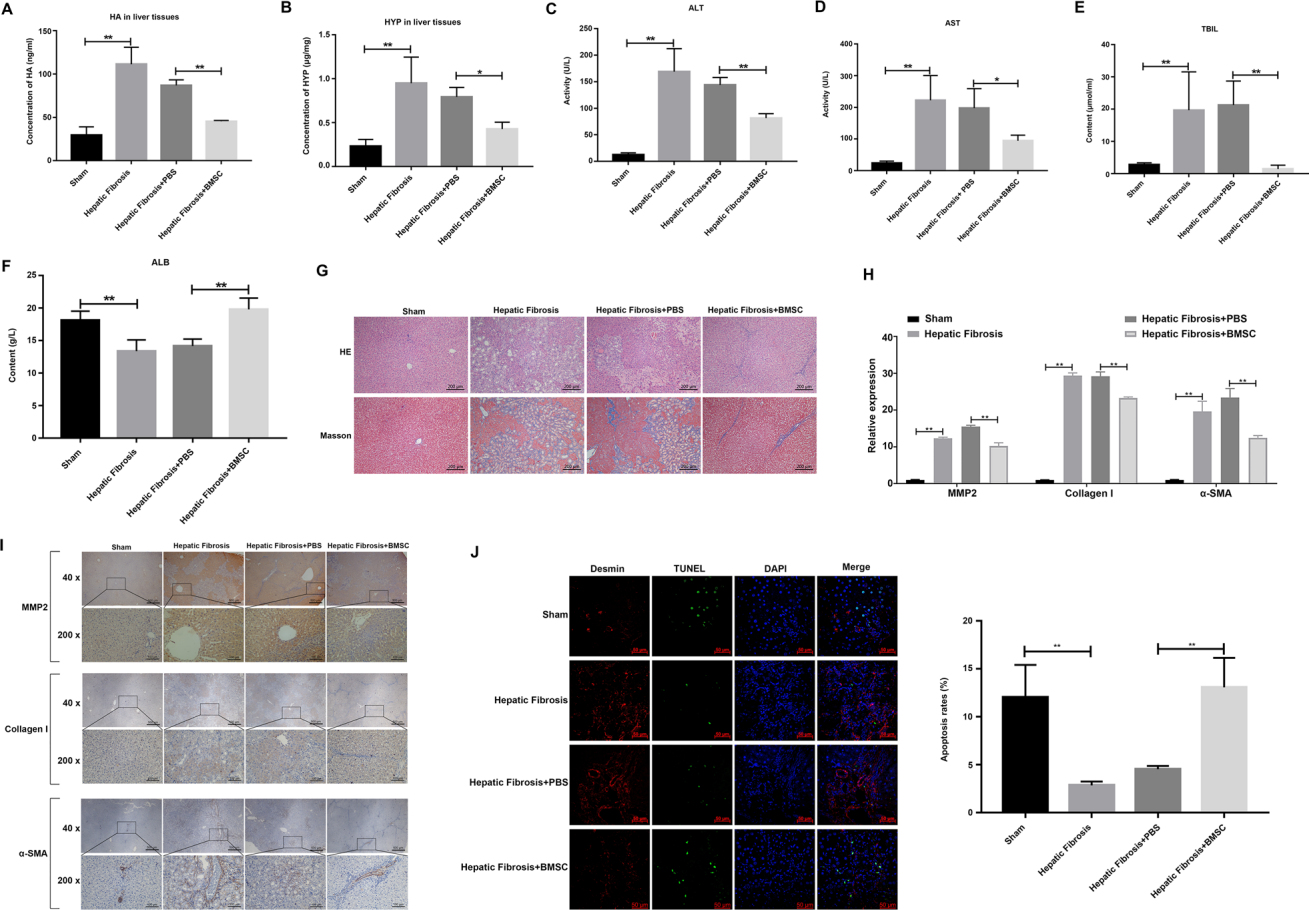
BMSCs alleviate hepatic fibrosis in a rat model. The concentration of HA (**A)**, HYP (**B)** in the liver tissues. The concentration of ALT (**C**), AST (**D**), TBIL (**E**), and ALB (**F**) in the serum of hepatic fibrosis rats treated with PBS or BMSCs was shown. **G** H&E and Masson’s staining showing liver microstructure of the collagen deposition in liver tissue of BMSC-treated and PBS-control rats. Magnification, 100×. qPCR (**H**) and IHC (**I**) showing the relative mRNA abundance and protein level of ECM-related genes in the liver tissue of rats. Magnification, 40× and 200×. **J** TUNEL and desmin colocalization staining showing apoptosis of desmin-positive cells in liver tissue after BMSC treatment. Magnification, 400×, scale bar = 50 μm

To investigate the effects of BMSCs on hepatic fibrosis rats, rats were first induced with CCl_4_ and treated with BMSCs. Hyp and HA contents in liver tissues, and serum ALT, AST, and TBIL was significantly upregulated in CCl_4_-induced rats (the hepatic fibrosis group), whereas BMSC injection (the hepatic fibrosis + BMSC group) significantly reversed the upregulation of these indicators caused by CCl_4_ (Fig. 2A–E). In contrast, the serum level of ALB was significantly reduced in the hepatic fibrosis group, which was reversed by BMSC treatment (Fig. 2F). BMSCs also alleviate the structural damage to the liver. H&E and Masson staining showed that collagen deposition in liver tissue was significantly upregulated in the hepatic fibrosis rats and was downregulated in the presence of BMSC treatments. As shown in Fig. 2G, the liver lobule structure in the rats of the sham group is clear and complete, hepatocyte cords are arranged radially from the central vein to the surroundings, and dilated hepatic sinusoids and collagen fibers were not observed; also, inflammatory cell infiltration or collagen fiber proliferation was not found. Compared with sham rats, the rats’ liver lobules in the liver tissue of hepatic fibrosis rats and those with hepatic fibrosis with PBS treatment were disordered, the structure of liver cells was destroyed, the fibrous connective tissue was proliferated, and most of the hepatocytes showed lamella necrosis, forming a typical pseudo-lobular structure of different sizes. The pseudo-lobules were mainly filled with fatty liver cells, with an increase in necrotic cells. However, liver injury was alleviated in BMSC-treated rats. Compared with the hepatic fibrosis rats, the liver lobule-like structure in the liver is normal, and there is not much collagen fiber proliferation.

In addition to studying the effect of BMSCs on pathological liver changes, we used qRT-PCR and IHC to detect the expression of fibrosis markers in the livers of rats with or without BMSC injection treatment. Both qRT-PCR and IHC results showed that the expression of MMP2, α-SMA, and collagen I in the liver tissue of fibrotic rats and PBS-treated fibrotic rats increased significantly compared with the sham group but was partially downregulated in the BMSC treatment group (Fig. 2H and I). Desmin was used as a marker to identify HSCs [21]. The TUNEL and desmin colocalization assay further showed that BMSC treatment significantly increased the apoptotic rate of desmin-positive cells in the liver tissue of the rats (Fig. 2J).

### AABR07028795.2 suppresses the TGF-β1-induced fibrotic phenotype of HSC-T6 cells

**Fig. 3:**
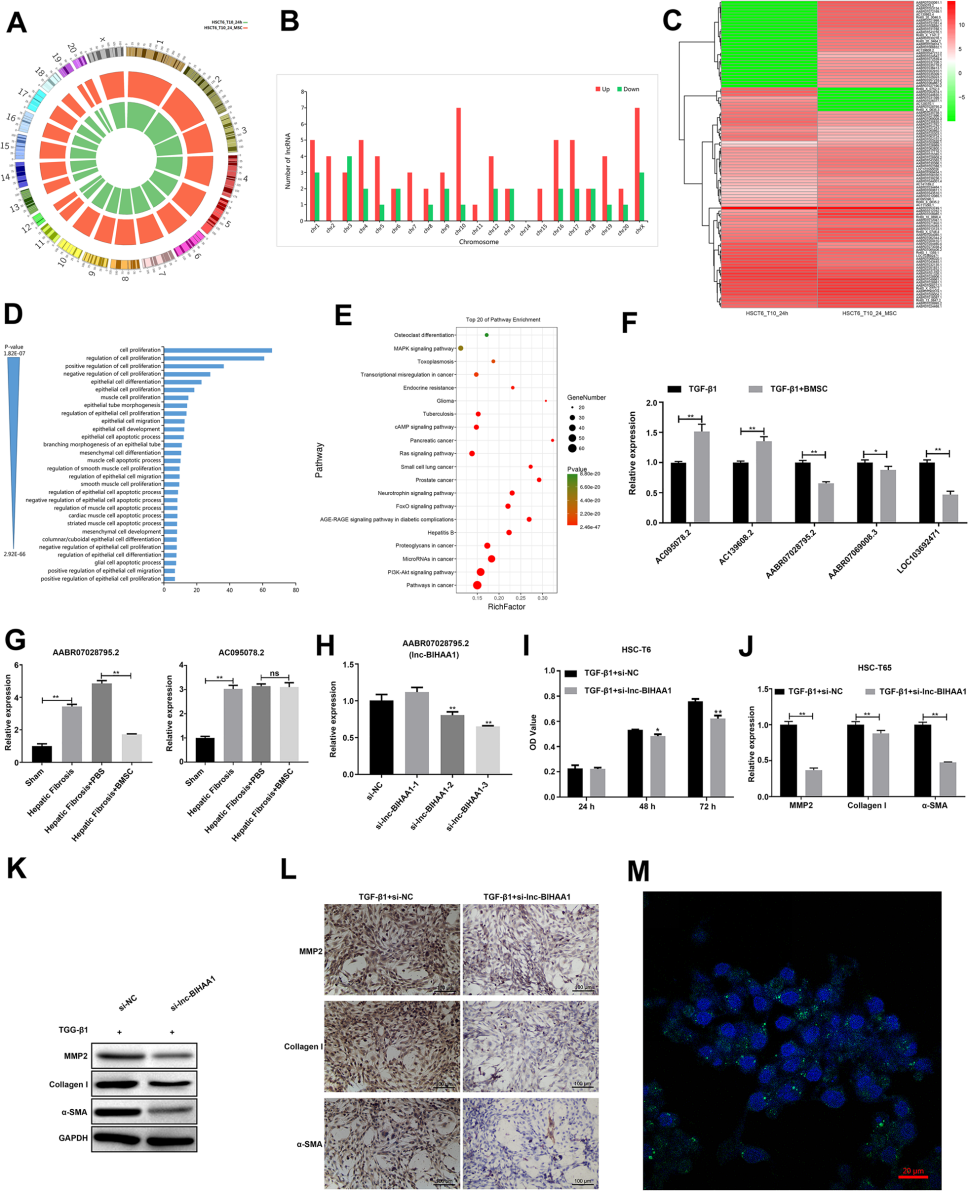
Knockdown of lnc-BIHAA1 suppresses the activation of HSC-T6 induced by TGF-β1. **A** The graph shows the density of each chromosome that total mapped reads compared with the genome. **B** Distribution of differentially expressed lncRNA on chromosomes. **C **The heat map of differentially expressed lncRNA. GO (**D**) and KEGG (**E**) analysis shows the possible function and pathways affected by the differentially expressed lncRNAs. **F** The differentially expressed lncRNAs’ response to the TGF-β1 induction was verified by qRT-PCR. **G** qRT-PCR shows the expression lncRNA response to BMSC treatment in the hepatic fibrosis rats. **H** qRT-PCR verifies the knockdown of lnc-BIHAA1 by siRNAs. **I** CCK-8 assay showing the effect of lnc-BIHAA1 siRNA on the activity of TGF-β1 inducted HSC-T6 cells. qPCR (**J**), WB (**K**), and IHC (**L**) assay showing the effect of lnc-BIHAA1 siRNA on the expression of fibrosis markers in TGF-β1-induced HSC-T6 cells. Magnification, 200×. **M** FISH assay showing the cellular location of lnc-BIHAA1 in HSC-T6 cells

To study the molecular mechanism of the inhibitory effect of BMSCs involved in hepatic fibrosis, TGF-β1-induced HSC-T6 cells with or without BMSC treatment were subjected to RNA-seq for analyzing the expression of lncRNAs in HSC-T6 in response to BMSC treatment. As shown in Fig. 3, RNA-seq results showed that all lncRNAs aligned to the genome were distributed on all chromosomes (Fig. 3A). Further analysis of the distributions of differentially expressed lncRNA showed that the upregulated lncRNAs were distributed on all chromosomes except chr14. The downregulated lncRNA is distributed on chromosomes, except for chr2, chr7, chr11, chr14, and chr15 (Fig. 3B). Using heat maps to visualize the expression profile of lncRNA found that a total of 103 lncRNA expressions changed significantly, of which 72 lncRNA expressions were upregulated and 31 lncRNA expressions were downregulated (Fig. 3C). Overall, the expression patterns of lncRNA in BMSC-treated cells were different from those without BMSC treatment. To predict the potential function of these lncRNAs, GO and KEGG analysis was performed. As shown in Fig. 3, GO analysis shows that the differentially expressed lncRNA is involved in cell apoptosis, cell proliferation, and EMT (fibrosis) transformation of GO term (Fig. 3D). KEGG enrichment analysis showed that differentially expressed lncRNA is mainly involved in the PI3K-AKT, FoxO, Ras, cAMP, and MAPK signaling pathways (Fig. 3E).

**Fig. 4:**
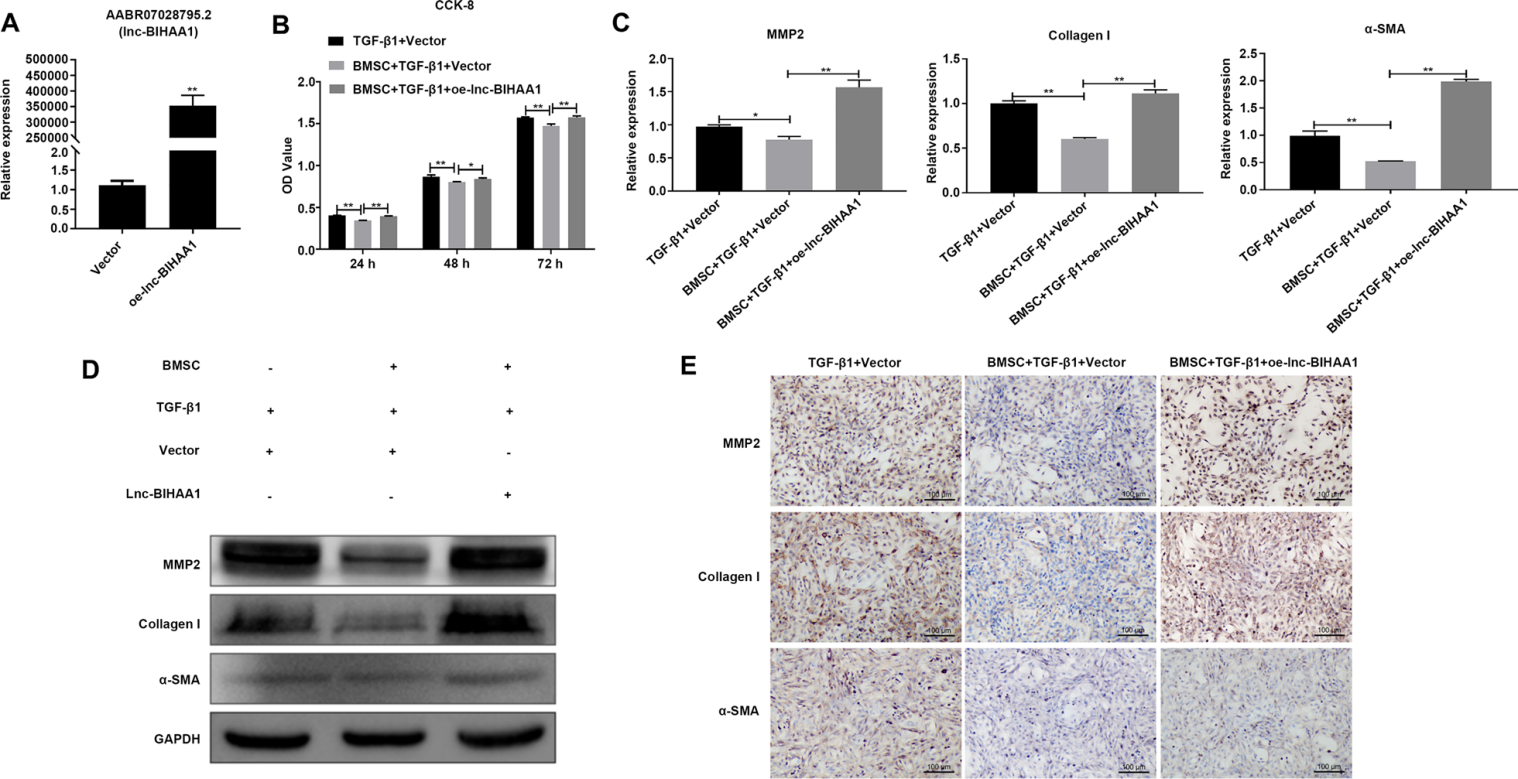
Lnc-BIHAA1 attenuated the beneficial effect of BMSCs on the activation of HSC-T6 induced by TGF-β1. **A** The overexpression efficiency of the lnc-BIHAA1 vector. Vector, empty vector without lnc-BIHAA1 fragment. **B** CCK-8 showed the effect of lnc-BIHAA1 overexpression on the activity of TGF-β1-induced HSC-T6 cells with or without BMSCs. qPCR (**C**), WB (**D**), and IHC (**E**) assay showing the effect of lnc-BIHAA1 overexpression on the expression of fibrosis markers in TGF-β1-induced HSC-T6 cells with or without BMSCs. Magnification, 200×

To verify the abundance of differentially expressed lncRNA found in RNA-seq, five of the top 10 differentially expressed lncRNAs with a length between 650 and 2000 bp, FDR < 0.05, and specific fragment regions were verified using qPCR in TGF-β1-induced HSC-T6 cells with or without BMSC treatment. Consistent with the RNA-seq results, the qPCR results showed that, of the five lncRNAs, AC095078.2, AC139608.2, AABR07028795.2, AABR07069008.3, and LOC103692471, which were differentially expressed in BMSC-treated cells (Fig. 3F), we selected two lncRNAs that are significantly upregulated and downregulated for follow-up experiments. The results showed that only AABR07028795.2 (namely lnc-BIHAA1) was also downregulated in BMSC-treated fibrotic rats (Fig. 3G). lnc-BIHAA1 was chosen for subsequent functional analysis.

To analyze the function of lnc-BIHAA1 in vitro, HSC-T6 cells first interfered with lnc-BIHAA1 siRNAs. qPCR results showed that si-lnc-BIHAA1-3 has the most evident interference effect among the three lnc-BIHAA1 siRNAs (Fig. 3H). CCK-8 assay results showed that compared with siNC, si-lnc-BIHAA1 transfection significantly reduced the increase in cell viability induced by TGF-β1 at 48 and 72 h after siRNA transfection (Fig. 3I) and suppressed the fibrosis markers, MMP2, collagen I, and α-SMA mRNA (Fig. 3J), and protein (Fig. 3K and L) expression. FISH results revealed that lnc-BIHAA1 is mainly located in the cytoplasm (Fig. 3M).

In contrast, overexpressed lnc-BIHAA1 (Fig. 4A) reversed the BMSC inhibition effect on the HSC-T6 activity, enhanced by the TGF-β1 treatment (Fig. 4B). Simultaneously, the inhibitory effect of BMSCs on MMP2, collagen I, α-SMA mRNA (Fig. 4C), and protein (Fig. 4D and E) was also reversed.

### *lnc-BIHAA1 suppresses hepatic fibrosis *via* targeting rno-miR-665*

**Fig. 5:**
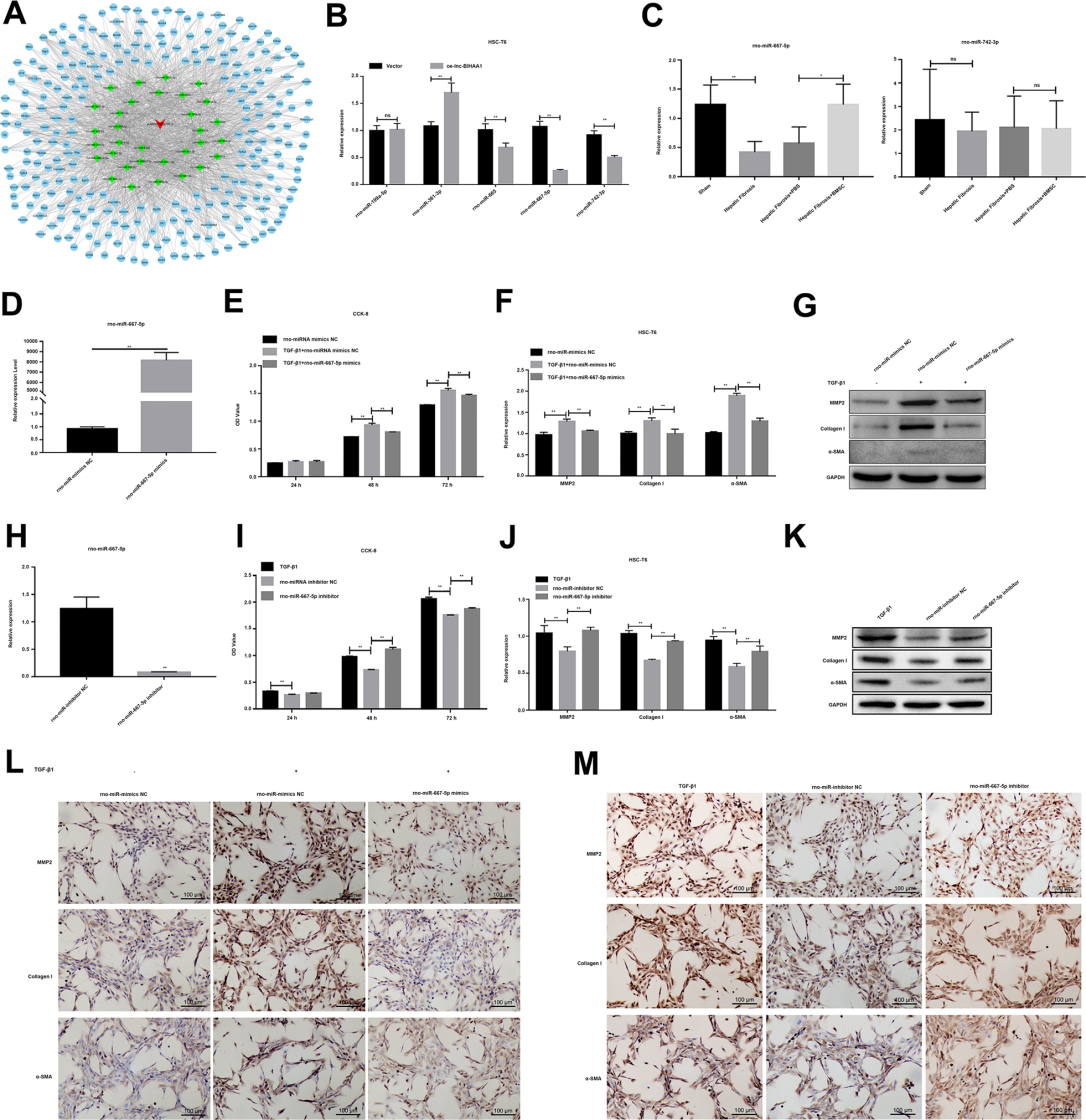
Rno-miR-667-5p inhibits fibrosis in HSC-T6 cells. **A** lnc-BIHAA1-miRNA interaction network. **B** qRT-PCR showing the five dysregulated miRNAs targeting lnc-BIHAA1 in the lnc-BIHAA1 overexpressing HSC-T6 cells. **C** qRT-PCR showing the expression of miRNAs with the most upregulated and downregulated fold change in hepatic fibrosis rats. **D** qRT-PCR showing the overexpression of rno-miR-667-5p mimics in HSC-T6 cells. **E** CCK-8 assay showing the effect of rno-miR-667-5p overexpression on the activity of TGF-β1 induces HSC-T6 cells. Expression of fibrosis-associated genes detected by qRT-PCR (**F**), WB (**G**), and IHC (**L**). Magnification, 200×. **H** qRT-PCR showing the inhibitory deficiency of rno-miR-667-5p inhibitors. **I** CCK-8 assay showing the effect of rno-miR-667-5p suppression on the activity of HSC-T6 cells. Expression of fibrosis-associated genes detected by qRT-PCR (**J**), WB (**K**), and IHC (**M**). Magnification, 200×

The ceRNA mechanism that targets miRNA and affects miRNA function is a molecular mechanism by which lncRNA regulates biological functions. To find the potential target miRNAs of lnc-BIHAA1, we built lnc-BIHAA1 as the center point and used the miRNAs, predicted by bioinformatics as a secondary connecting node to construct the lnc-BIHAA1-miRNA interaction network (Fig. 5A). According to the number of binding sites, five key miRNAs were selected for further expression verification. The qPCR results showed that the expressions of rno-miR-665, rno-miR-667-5p, and rno-miR-742-3p were significantly decreased in HSC-T6 cells after lnc-BIHAA1 overexpression, which matches the ceRNA regulation mode (Fig. 5B). Among them, rno-miR-667-5p is downregulated in liver tissue samples of hepatic fibrosis rats (Fig. 5C), suggesting that rno-miR-667-5p is a downstream molecule regulated by lnc-BIHAA1.

To answer whether rno-miR-667-5p is involved in regulating liver fibrosis, first, HSC-T6 cells were transfected with rno-miR-667-5p mimic and rno-miR-667-5p inhibitor to overexpress and inhibit rno-miR-667-5p expression (Fig. 5D and H). Then, the cells were stimulated with TGF-β1. CCK-8 assay results showed that rno-miR-667-5p mimic significantly inhibited the increase in cell viability induced by TGF-β1 treatment for 48 and 72 h (Fig. 5E), while rno-miR-667-5p inhibitor was opposite to the rno-miR-667-5p mimic (Fig. 5I). qPCR results show that miR-667-5p mimic inhibits TGF-β1-induced mRNA (Fig. 5F) and protein expression (Fig. 5G and L) of MMP2, collagen I, and α-SMA in HSC-T6. In contrast, miR-667-5p inhibitor promoted the mRNA (Fig. 5J) and protein expression (Fig. 5K and M) of these genes.

**Fig. 6:**
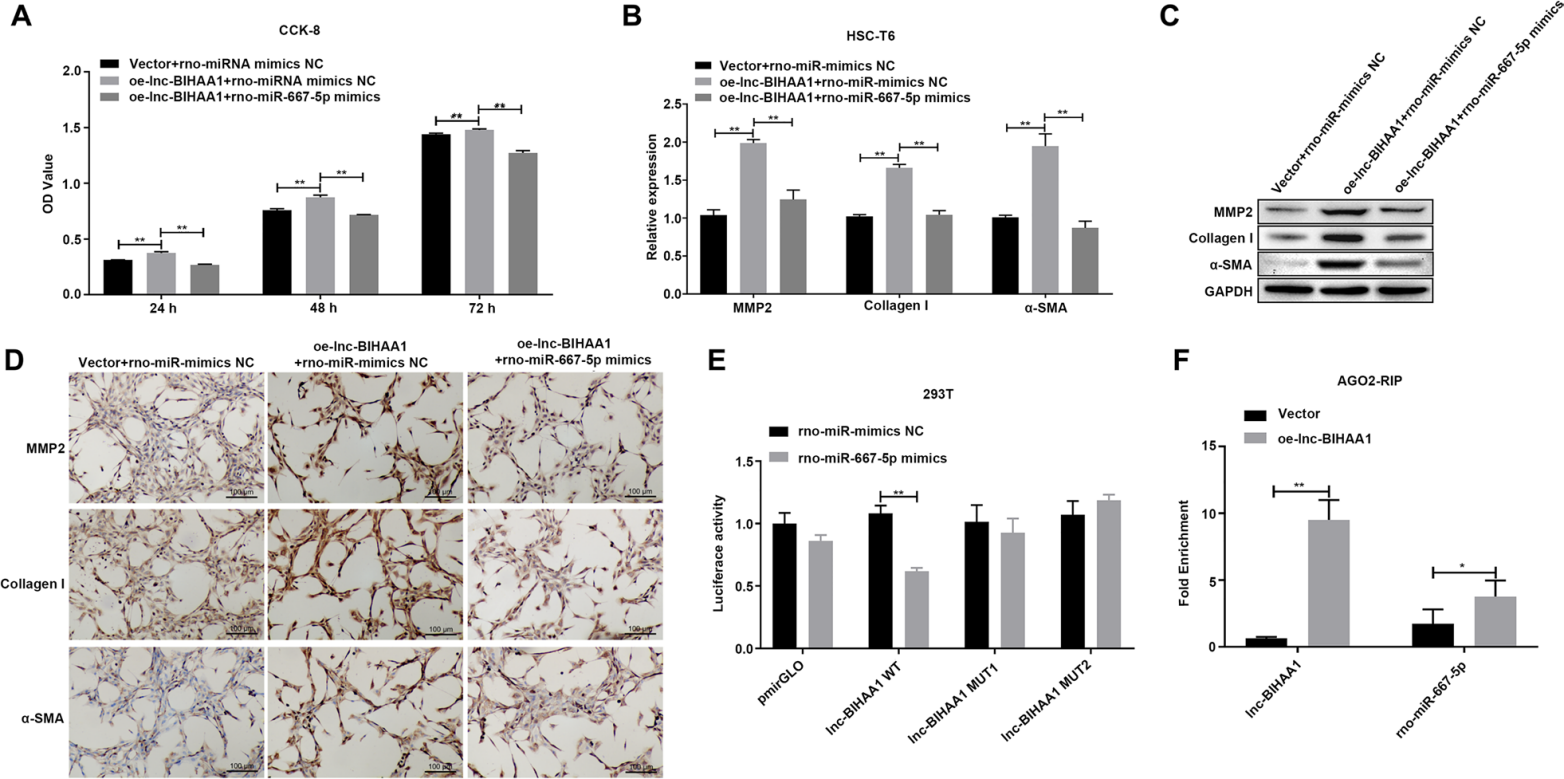
Lnc-BIHAA1 binds and suppresses rno-miR-665 expression to promote liver fibrosis in HSC-T6 cells. **A** CCK-8 assay showing the inhibitory effects of rno-miR-667-5p mimics on cell activity in lnc-BIHAA1 overexpressing cells. The relative expression of fibrosis-associated genes mRNA and protein detected by qPCR (**B**), WB (**C**), and IHC (**D**). Magnification, 200×. Luciferase activity assay (**E**) and RIP assay (**F**) show the interaction between lnc-BIHAA1 and rno-miR-665

To explore the relationship between rno-miR-667-5p and lnc-BIHAA1, rno-miR-667-5p mimic was transfected into lnc-BIHAA1 overexpressing HSC-T6 cells. As shown in Fig. 6, rno-miR-667-5p mimic significantly inhibited the increase in HSC-T6 cell viability at 24, 48, and 72 h post-transfection (Fig. 6A) and inhibited MMP2, collagen I, and α-SMA mRNA (Fig. 6B) and protein expression (Fig. 6C and D). Luciferase assay revealed that only the luciferase activity of the vector containing WT lnc-BIHAA1 sequence was inhibited by rno-miR-667-5p mimics, while the luciferase activity of vector harboring MUT lnc-BIHAA1 did not change significantly (Fig. 6E). Further RIP assay verified that significantly increased rno-miR-667-5p was observed in the Ago2 complex in cells overexpressing lnc-BIHAA1, suggesting increased recruitment of rno-miR-667-5p into RNA-induced silencing complex in the case of lnc-BIHAA overexpression (Fig. 6F).

**Fig. 7:**
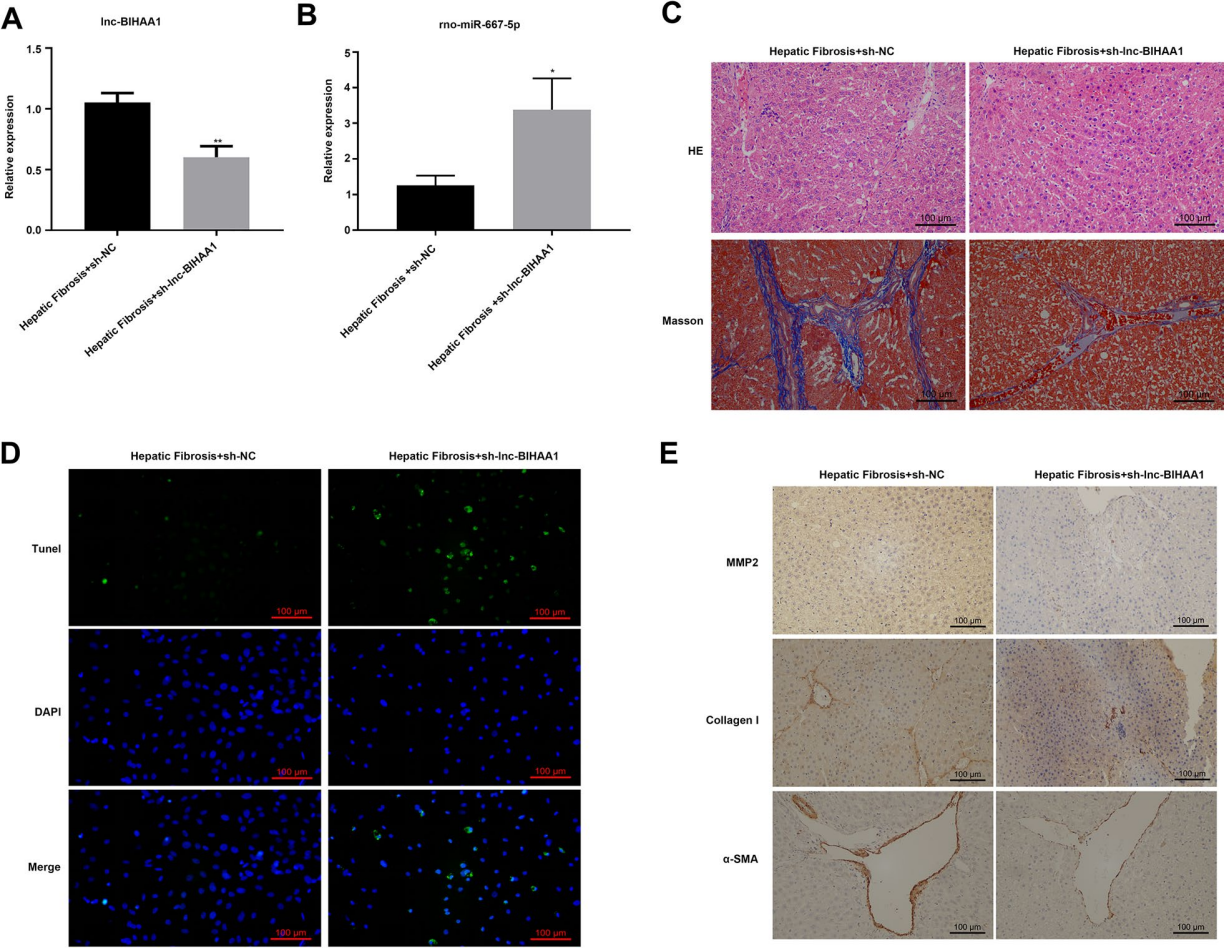
Knockdown of lnc-BIHAA1 using sh-lnc-BIHAA1 lentivirus attenuated hepatic fibrosis in rats. qRT-PCR showing the expression of lnc-BIHAA1 (**A**) and its target rno-miR-667-5p (**B**) in sh-lnc-BIHAA1-infected hepatic fibrosis rats. **C** H&E and Masson staining show the recovery of histology and fibrosis caused by sh-lnc-BIHAA1 lentivirus. Magnification, 200×. **D** TUNEL assay showing cell apoptosis in sh-lnc-BIHAA1-infected hepatic fibrosis rats. Magnification, 200×. **E** IHC showing the expression of fibrosis-associated proteins expression. Magnification, 200×

The qRT-PCR results showed that the expression of lnc-BIHAA1 was significantly downregulated in rats injected with sh-lnc-BIHAA1 (Fig. 7A), whereas rno-miR-667-5p was significantly upregulated (Fig. 7B). The results of H&E and Masson staining showed that inhibiting the expression of lnc-BIHAA1 attenuated liver fibrosis: the liver cell structure in the liver tissue of the sh-NC group was destroyed, and the collagen fibers were prominent, whereas in the liver tissue of the sh-lnc-BIHAA1 group, the morphology of liver cells was normal, the lobular structure of the liver was normal compared with the sh-NC group, and the hyperplasia of collagen fibers was less (Fig. 7C). TUNEL assay results showed that the apoptosis of liver tissues in the sh-lnc-BIHAA1 group was significantly increased compared with that of the sh-NC group (Fig. 7D). In addition, the expression of MMP2, collagen I, and α-SMA protein in liver tissue was significantly reduced by IHC (Fig. 7E) in the sh-lnc-BIHAA1 group. The results showed that compared with the sh-NC group, the expression of ECM-related proteins in the sh-lnc-BIHAA1 group were significantly reduced.

## Discussion

This study found the anti-fibrotic effects of BMSC treatment on TGF-β1-induced HSC-T6 cells and CCl_4_-induced hepatic fibrosis rats. A further mechanism study revealed that lnc-BIHAA1, which was downregulated by BMSC treatment, suppressed hepatic fibrosis by binding to rno-miR-667-5p and upregulated the expression of rno-miR-667-5p to inhibit fibrosis.

HSC is the primary source of ECM that contributes to fibrosis significantly. Overproduction of ECM is the primary pathological feature of hepatic fibrosis [22]. HSC is in quiescent status in the normal liver, and the production and degradation of ECM are balanced. However, when the HSC is activated into a myofibroblast-like phenotype by inflammation or cytokines, the ECM is overexpressed, and the expression of α-SMA is upregulated [2] and leads to the distortion of normal liver architecture. TGFβ has been proven as a pro-fibrotic cytokine. Notably, HSC activation is supported by TGF-β [23]. TGF-β1 can promote and maintain the expression of collagen genes and affects resisting HSC cell apoptosis [24]. In this study, we used TGF-β1 to induce HSC-T6 cells. As expected, cell activation of HSC6 was increased, indicating an increase in cell proliferation of HSC. Also, increased ECM and myofibroblast-like phenotype were observed in the cells, which indicated the activation of HSCs and acquisition of the pro-fibrosis phenotype.

BMSC treatment has been suggested to be a promising therapy for fibrosis. However, the underlying mechanism still needs to be investigated. lncRNA has the function of regulating liver fibrosis. lncRNA mainly enhances the pyroptosis of macrophages, inhibits the classic pro-fibrosis pathway, and inhibits HSC activation to alleviate liver fibrosis [25]. Although lncRNA related to liver fibrosis has been reported in many studies, as reported in the literature review [26]. However, the regulatory function of a large number of lncRNAs in liver fibrosis and whether the effect of BMSCs is associated with lncRNA remain to be studied. In our study, we found that lnc-BIHAA1 has the function of promoting liver fibrosis. As of our best knowledge, few studies have been reported regarding the function of lnc-BIHAA1. Thus, our research has increased the understanding of lncRNA in regulating liver fibrosis. We also suggest that lnc-BIHAA1 is one of the effector molecules of BMSC therapy.

miRNA is involved in regulating the formation and progression of liver fibrosis. Both endogenous and extracellular vesicles have the role of repairing injured tissue and suppressing fibrosis [27–29]. Of the miRNAs, microRNA-29b is an important regulator of liver fibrosis. Many studies have revealed that microRNA-29b regulates the level of gene methylation modification and inhibits liver fibrosis by inhibiting HSC cell activation [30–32]. Also, microRNA-17-5p, microRNA-222, and microRNA-221-3p have been shown to promote fibrosis [33–35], and microRNA-378 and microRNA-101 inhibit HSC activation in liver fibrosis [36, 37]. We found that rno-miR-667-5p inhibited the increase in HSC-T6 cell viability caused by the expression of lnc-BIHAA1 and inhibited the expression of fibrosis markers. As there is still a lack of extensive research on the function of rno-miR-667-5p, our research also suggests new molecules for miRNA to regulate liver fibrosis. In addition, our results also advanced the role of miRNA in BMSC treatment for live fibrosis.

Although the mechanism of lncRNA in regulating gene expression has not been fully understood, the function of lncRNA can vary depending on the cell location. Evidence has shown that lncRNA can bind RNA and protein [38]. For example, the latest research has shown that lncRNA in the nucleus has the function of preventing nuclear target molecules that regulate RNA stability from shuttling into the cytoplasm, thereby affecting RNA stability [39]. In the cytoplasm, emerging evidence has proven that lncRNA functions as an endogenous RNA (ceRNA) to sponge miRNA through the miRNA response elements [40, 41]. By adsorbing miRNA, lncRNA effectively inhibits the miRNA target expression and function downstream [42, 43]. Recently, many studies have shown that lncRNAs act as ceRNAs to target downstream miRNAs. Of these ceRNA mechanisms, LincRNA-p21 /MicroRNA-17-5p, lncRNA GAS5/miR-23a, LncRNA Gm5091/miR-27b/23b/24, and MALAT1/miR-101b have been suggested to contribute to the alleviation of liver fibrosis [15–18, 44]. Our study found that lnc-BIHAA1 is mainly located in the cytoplasm and can be combined with rno-miR-667-5p. Further analysis found that rno-miR-667-5p can reverse the promoting effect of lnc-BIHAA1 on the fibrous phenotype of HSC-T6 at the cellular level. In addition, animal experiments also found that lnc-BIHAA1 in rat fibrotic liver tissue was downregulated after BMSC treatment, whereas rno-miR-667-5p was upregulated. These results suggest that the interaction of lnc-BIHAA1 and rno-miR-667-5p has an anti-fibrosis effect.

RNA-induced silencing complex is a complex for gene silencing. RNA interference is triggered by signals, such as dsRNA and ribonucleoprotein. For example, Argonaute 2, a key component of RNA-induced silencing complex, binds to small regulatory RNA and is guided to target downstream transcripts [45]. This study first found the potential biding site of rno-miR-667-5p and lnc-BIHAA1 using luciferase assay. Mainly, rno-miR-667-5p and lnc-BIHAA1 was observed in the AGO2 immunoprecipitated complex, and the abundance of rno-miR-667-5p was increased, accompanied by lnc-BIHAA1 expression. These results suggest that lnc-BIHAA1 upregulation promotes the recruitment of rno-miR-667-5p into the RNA-induced silencing complex, guided by RNA and silencing gene expression [46]. Combining the different functions of rno-miR-667-5p and lnc-BIHAA1 in liver fibrosis and their interactions, we believe that BMSC therapy may exert anti-fibrotic effects by regulating the lnc-BIHAA1/rno-miR-667-5p axis.

## Conclusions

We found that BMSCs alleviate liver fibrosis, inhibit the activation of HSC induced by TGF-β1, and promote apoptosis. One possible mechanism is to inhibit the expression of lnc-BIHAA1, thereby inhibiting its binding to downstream rno-miR-667-5p and enhancing the anti-fibrosis function of rno-miR-667-5p. Our research provides a molecular mechanism of BMSCs in treating liver fibrosis and provides a theoretical basis for promoting the clinical application of BMSCs in treating liver fibrosis.

## Data Availability

The data used to support the findings of this study are available from the corresponding author upon request.

## Abbreviations

BMSCs: Bone marrow mesenchymal stem cells
ECM: Extracellular matrix
HSCs: Hepatic stellate cells
CCK-8: Cell counting kit-8
WB: Western blotting
qRT-PCR: Real-time quantitative PCR
HE: Hematoxylin and eosin
RNA-seq: RNA sequencing
GO: Gene Ontology
KEGG: Kyoto Encyclopedia of Genes and Genomes
TUNEL: Transferase dUTP nick end labeling
FISH: Fluorescent in situ hybridization
HA: Hyaluronic acid
HYP: Hydroxyproline
WT: Wild type
MUT: Mutant
RIP: RNA immunoprecipitation
SD: Standard deviation
